# ATF4 over-expression increased IgG_1_ productivity in Chinese hamster ovary cells

**DOI:** 10.1186/1753-6561-5-S8-O11

**Published:** 2011-11-22

**Authors:** Ahmad M Haredy, Akitoshi Nishizawa, Kohsuke Honda, Tomoshi Ohya, Hisao Ohtake, Takeshi Omasa

**Affiliations:** 1 Department of Biotechnology, Graduate School of Engineering, Osaka University, Suita, Osaka, 565-0871, Japan; 2 Tanabe Mitsubishi Pharma, Hirakata, Osaka, Japan, 573-1153; 3 Institute of Technology and Science, The University of Tokushima, Tokushima, 770-8506, Japan

## Introduction

The endoplasmic reticulum (ER) is the major organelle of synthesis of protein and forms a membranous network throughout the cell. According to Shimizu and Hendershot [1], about one third of the total proteins produced are synthesized in the ER. The ER lumen possesses a unique environment for high quality control for protein folding and assembly. It contains high concentrations of molecular chaperones, folding enzymes, and ATP, which aid in proper maturation of proteins [2]. However, if the amount of proteins to be folded exceeds the capacity of the folding machineries, unfolded proteins will accumulate in the ER and Unfolded protein response (UPR) will start. UPR aims at regaining the homeostasis inside the ER by attenuating the protein translation, activating the folding machineries, degradation of the unfolded proteins, and finally apoptosis. Activating transcription factor 4 (ATF4) is a central factor in the UPR pathways which is involved in folding and processing of secretory proteins. Our previous research showed that *ATF4* over-expression is efficient for increasing the productivity of antithrombin-III [3,4]. In this study, we investigated if this approach is product-specific or not.

## Materials and methods

### Cell line and medium

Serum-free adapted Chinese hamster ovary DP-12-SF (CHO DP-12-SF) cell line (producing human anti-IL-8 IgG_1_) and Dulbecco Modified MEM medium supplemented with 10% dialyzed fetal bovine serum (FBS) and 200nM methotrexate were used in this study.

### Vector construction

The *ATF4* expression plasmid was constructed using CHO ATF4**cDNA into *Kpn*I/*Xba*I site of the pcDNA3.1/Hygro(+) expression vector (Invitrogen, Carlsbad, CA, USA), designated as pcDNA3.1/Hygro(+)-ATF4.

### Transfection and selection

*The* pcDNA3.1/Hygro(+)-ATF4 vector was transfected into CHO DP-12-SF cell line using a TransIT-LT1 transfection reagent (Mirus bio Madison, WI USA). Single cell clones were obtained using the limiting dilution method. The transfected cell lines were selected using 200μg/ml hygromycin.

### Chromosomal integration of ATF4

Genomic DNA was isolated after 72 h of cultivation using DNeasy blood and tissue extraction kit (Qiagen, Chatsworth, CA, USA). The primers 5`-TAATACGACTCACTATAGGG-3` and 5`-TAGAAGGCACAGTCGAGG-3` were employed for the amplification of non-coding region of pcDNA3.1/Hygro(+)-ATF4 for detection of the integration of the designated plasmid into the CHO chromosome. PCR was performed using *Ex Taq* polymerase (Takara Bio, Otsu, Shiga JAPAN) with 100 ng of the genomic DNA.

### Confirmation of ATF4 expression

RNA was isolated after 72 h of cultivation using RNeasy mini kit (Qiagen). cDNA equivalent to 1μg of the previously prepared RNA was prepared using omniscript RT kit (Qiagen) and PCR was performed using the same primers for chromosomal detection.

### Cell and antibody concentrations

Cell concentration was determined using Coulter Vi-Cell Automated Cell Viability Analyzer (Beckman Coulter, Inc., Fullerton, CA, USA). Antibody concentration was determined by sandwich enzyme linked immunosorbent assay (ELISA) [5]. Kinetic parameters were calculated as described previously [3].

*Real time PCR:* The quantification of mRNA of heavy and light chains of IgG was performed by the SYBR Green real-time RT-PCR method (Applied Biosystems, Foster City, CA, USA) using the StepOnePlus Real-Time PCR System.

## Results and discussion

The gene encoding *ATF4* was cloned from CHO-K1 inserted into the multiple cloning site of pcDNA3.1 vector with hygromycin cassette as a selection marker. The *ATF4* expression vector was then transfected into CHO DP-12-SF cell line [5], producing humanized anti IL-8 Immunoglobulin-1 (IgG_1_). Twenty six single cell clones were then established using the limiting dilution method. To examine if pcDNA3.1/Hygro(+)-ATF4 was integrated into the CHO chromosome or not, PCR analysis were performed using the primers designed for non-coding region of the expression vector. Only 5 clones were confirmed with the insert at 1.2 Kb; CHO DP-12-ATF4-3, -9, -10, -12, and-16. Reverse Transcriptase-Polymerase Chain Reaction analyses revealed that only 3 clones, CHO DP-12-ATF4-10, -12, and -16, showed positive *ATF4* expression. After 144 hours of cultivation, only clones with confirmed *ATF4* expression showed significant increase in specific IgG_1_ production rate ranging from 1.8 to 2.5 times the parental CHO DP-12-SF cell. Clone DP-12-ATF4-16 that showed the highest increase in specific productivity with about no change in the specific growth rate was subjected to further analysis for quantification of mRNA of heavy and light chains to determine if over-expression affects the transcription of mRNA or not. The result was found in agreement with our previous research that over-expression of *ATF4* did not significantly change the level of the product mRNA [4]. It suggested that *ATF4* over-expression might improve the translation and the secretion without affecting the transcription.

## References

[B1] Shimizu Y Hendershot LM Organization of the functions and components of the endoplasmic reticulum Adv Exp Med Biol 2007 594 37 46 10.1007/978-0-387-39975-1_4 17205673

[B2] Gomez E Powell ML Bevington A Herbert TP A decrease in cellular energy status stimulates PERK-dependent eIF2alpha phosphorylation and regulates protein synthesis in pancreatic beta-cells Biochem J 2008 410 485 493 10.1042/BJ20071367 18052927

[B3] Ohya T Hayashi T Kiyama E Nishii H Miki H Kobayashi K Honda K Omasa T Ohtake H Improved production of recombinant human antithrombin III in Chinese hamster ovary cells by ATF4 overexpression Biotechnol Bioeng 2008 100 317 324 10.1002/bit.21758 18078289

[B4] Omasa T Takami T Ohya T Kiyama E Hayashi T Nishii H Miki H Kobayashi K Honda K Ohtake H Overexpression of GADD34 enhances production of recombinant human antithrombin III in Chinese hamster ovary cells J Biosci Bioeng 2008 106 568 573 10.1263/jbb.106.568 19134553

[B5] Kim WD Tokunaga M Ozaki H Ishibashi T Honda K Kajiura H Fujiyama K Asano R Kumagai I Omasa T Ohtake H Glycosylation pattern of humanized IgG-like bispecific antibody produced by recombinant CHO cells Appl Microbiol Biotechnol 2010 85 535 542 10.1007/s00253-009-2152-z 19652963

